# Incidental operating room fire from a breathing circuit warmer system: a case report

**DOI:** 10.1186/s12871-021-01488-2

**Published:** 2021-11-05

**Authors:** Wangseok Do, Dahyun Kang, Purna Hong, Hyae-Jin Kim, Jiseok Baik, Dowon Lee

**Affiliations:** 1 Biomedical Research Institute, Pusan National University Hospital, Busan, 49241 Republic of Korea; 2 Department of Anesthesia and Pain Medicine, School of Medicine, Pusan National University, 179 Gudeok-ro, Seo-gu, Busan, 49241 Republic of Korea

**Keywords:** Operating room fire, Breathing circuit warmer, Anesthesia fire, Airway fire

## Abstract

**Background:**

An airway-associated fire in an operating room can have devastating consequences for patients. Breathing circuit warmers (BCWs) are widely used to provide heated and humidified anesthetic gases and eventually prevent hypothermia during general anesthesia. Herein, we describe a case of a BCW-related airway fire.

**Case presentation:**

In this case, an electrical short within a BCW wire caused a fire inside the circuit. Simultaneously, the fire was extinguished, ventilation was stopped, and the endotracheal tube was disconnected from the BCW. The patient was exposed to the fire for less than 10 s, resulting in burns to the trachea and bronchi. Immediately after airway burn, bronchoscopy showed no edema or narrowing except for soot in the trachea and both main bronchus. After the inhalation burn event, prophylactic antibiotics, bronchodilator, mucolytics nebulizer, and corticosteroid nebulizer were started. On bronchoscopy 3 days after the inhalation burn, mucosal erythematous edema was observed and the inflammatory reaction worsened. The inflammatory reaction showed aggravation for up to 2 weeks, and then gradually recovered, and the epithelium and mucous membrane of the upper respiratory tract returned to normal after 4 weeks. Eventually, the patient recovered without long-term complications and was successfully discharged.

**Conclusions:**

This is the first report of a fire caused by BCW. We wanted to share our experience of how we responded to an airway-related fire in an OR and treated the patient. It cannot be overemphasized that the electrical medical appliance associated with the airways are fatal to the patient in the event of a fire, so caution should always be exercised.

## Background

Operating room (OR) fires are dangerous events that occur at least 650 times annually [1]. Fires only occur when the three major factors are available: ignition source, oxygen, and fuel. All three factors exist routinely in OR. There are ignition sources such as electric cautery and surgical electric devices, oxidizers such as anesthetic gases supply, and fuels such as alcohol-containing skin disinfectants and lap pads [2]. In particular, during general anesthesia, the airway and breathing circuit from the anesthetic machine to the patient’s respiratory system satisfy all three factors of fire. As high concentration of oxygen is directly supplied to respiratory system of patients, fires around anesthesia machines, breathing circuits, and endotracheal tubes (ETTs) could be fatal for patients. A breathing circuit warmer (BCW, Mega Acer Kit®, ACE Medical, South Korea) supplies patients with heated and humidified anesthetic gases and prevent hypothermia during general anesthesia [3]. The authors experienced an airway fire associated with a BCW, which has not been previously reported. Therefore, we describe this rare occurrence and provide a review of the literature.

## Case presentation

A 93-year-old female patient (weight, 32 kg; height, 130 cm) was scheduled for general anesthesia for the reconstruction of a soft tissue defect on her left cheek. She had no remarkable medical history, except for well-controlled hypertension. Preoperative chest radiography was normal and pulmonary function tests revealed normal respiratory function.

Glycopyrrolate (0.2 mg) was administered intramuscularly for premedication 30 min preoperatively. In the OR a standard monitoring, including pulse oximetry, electrocardiography, and non-invasive blood pressure measurement, was applied. Baseline vital signs included a heart rate (HR) of 64 bpm; blood pressure (BP), 188/85 mmHg; and blood oxygen saturation (SpO_2_), 92% on room air. General anesthesia was induced intravenously, using 40 mg of propofol, followed by continuous administration of remifentanil (0.26 mcg/kg/min), and 30 mg of rocuronium. Oral endotracheal intubation was performed with a direct laryngoscope and a 6.0-mm reinforced ETT. An anesthesia machine supplied a gas mixture with a fraction of inspired oxygen (FiO_2_) of 50% at a total flow rate of 2 L/min. Anesthesia was maintained using 5.0 vol% desflurane and 0.1 mcg/kg/min of remifentanil. The tidal volume and respiratory rate were set to 200 ml and 14 per min, respectively. After induction of anesthesia was completed, the patient’s posture and position of the anesthesia machine were adjusted. In facial surgery, we routinely place the anesthesia machine on the side of the patient’s right lower limb; therefore, the breathing circuit is naturally placed on the neck, chest, and abdomen.

**Fig. 1:**
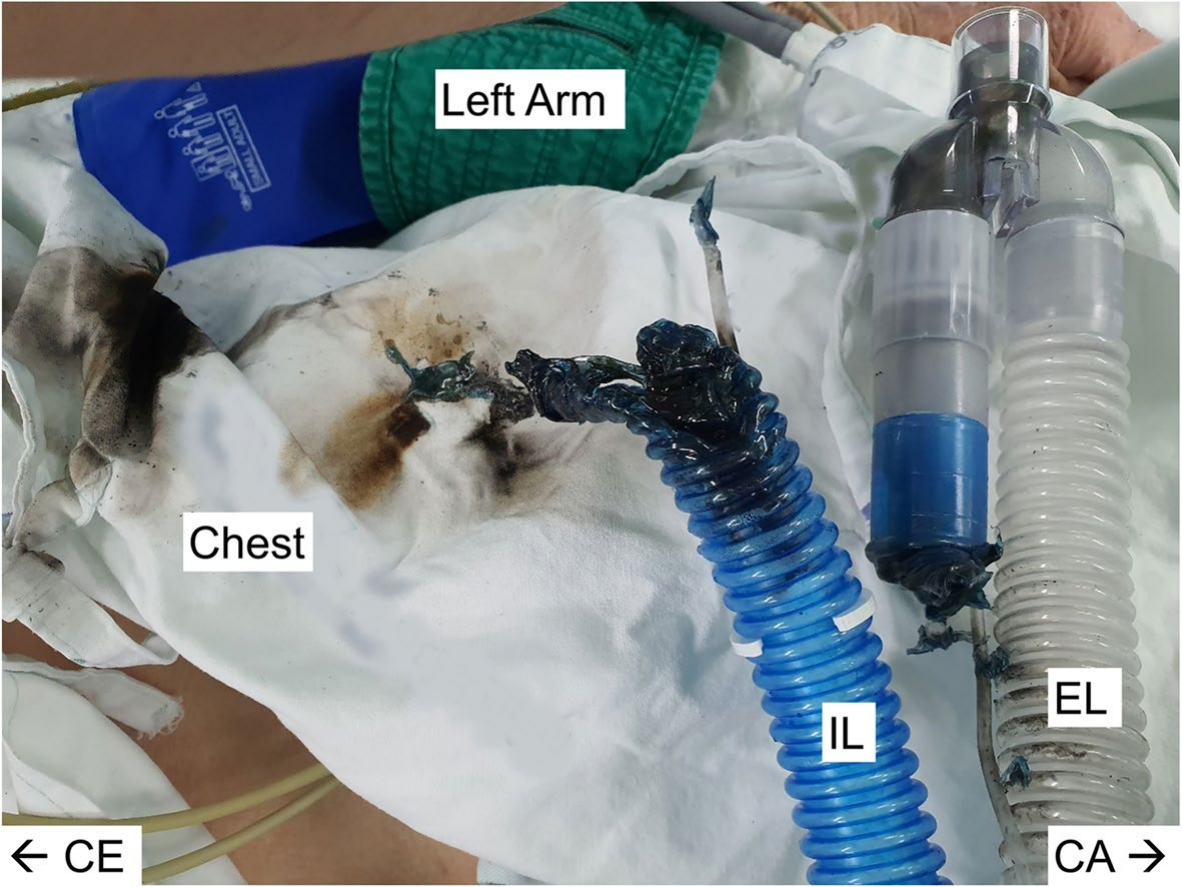
Breathing circuit warmer (BCW) and patient’s clothing. BCW and patient’s clothing immediately after extinguishing the fire. The patient’s end of the BCW is completely melted and separated, burn marks from a flame can be seen on the patient’s clothes. BCW is removed from the endotracheal tube and placed on the patient’s abdomen. Soot can be observed in the inspiratory limb of the respiratory circuit. IL inspiratory limb, EL expiratory limb, CE cephalad direction, CA caudad direction

**Fig. 2:**
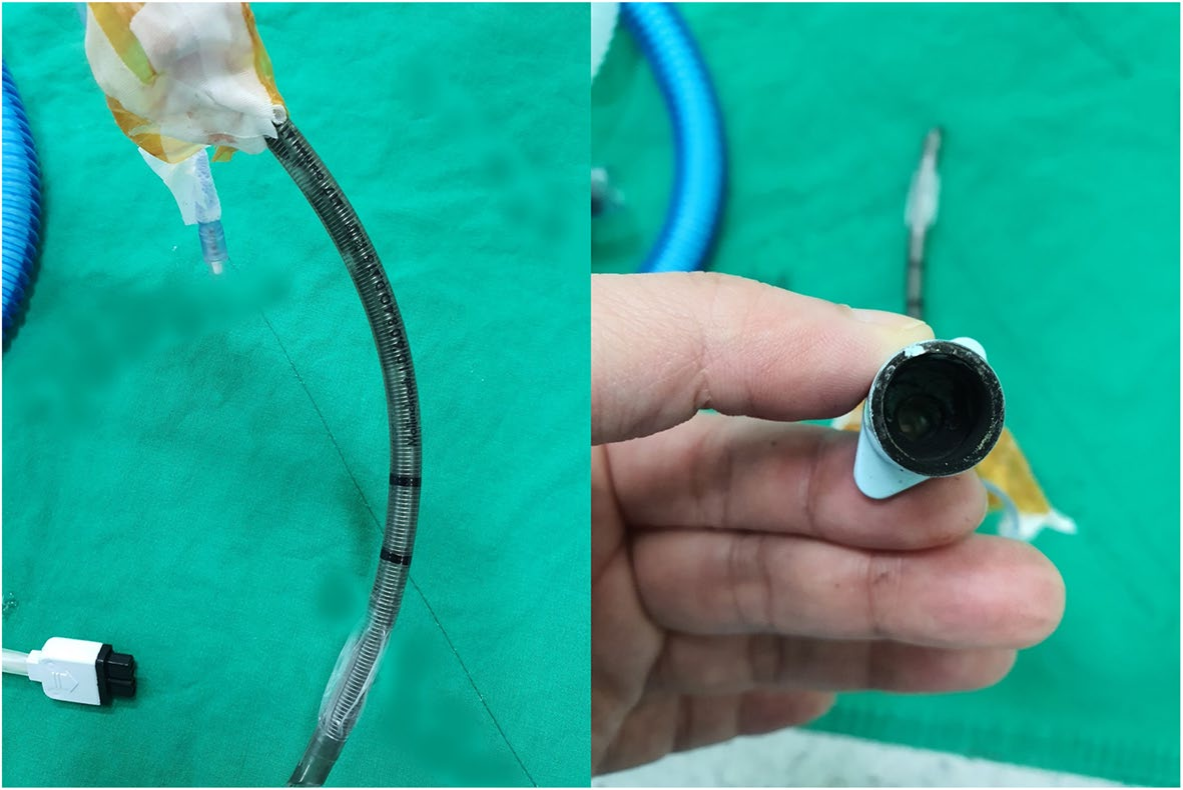
Endotracheal tube. Endotracheal tube with which the patient was intubated during the fire. Soot can be seen inside the tube

Given that the scheduled operation time was 6 h, a BCW was applied routinely to prevent hypothermia during anesthesia. Once surgical site preparation was almost complete, the BCW was turned on. Immediately, there was a spark with a popping sound, and smoke and flames could be seen at the patient’s end of the breathing circuit (Fig. 1). An anesthesiologist and an anesthesiology resident doctor were participating in induction of anesthesia. The anesthesiologist disconnected the ETT and the breathing circuit and turned off the anesthesia machine and gas supply, within 10 s of the fire starting. Anesthesiology resident doctor extinguished the flames on the patient’s clothing by pouring water. After the fire had been completely extinguished, ventilation was restarted with an Ambu bag with 100% oxygen. The vital signs at that moment were as follows: HR, 72 bpm; BP, 159/63 mmHg; body temperature, 36.5 °C; and SpO_2_, 100%. As there was a significant amount of soot inside the ETT (Fig. 2), we observed the upper airway using fiber-optic bronchoscopy (FOB). Soot was observed in the trachea and carina. The ETT was replaced with a new same-sized reinforced ETT using a video laryngoscope. After the fire, general anesthesia was switched from inhalation to total intravenous anesthesia and the surgery was suspended. At that time, the results of her arterial blood gas analysis were as follows: PH 7.401; PCO_2_, 49.5 mmHg; PO_2_ 202.9 mmHg; and SaO_2_, 99.7%. The patient was transferred to an intensive care unit (ICU) with stable vital signs.

**Fig. 3:**
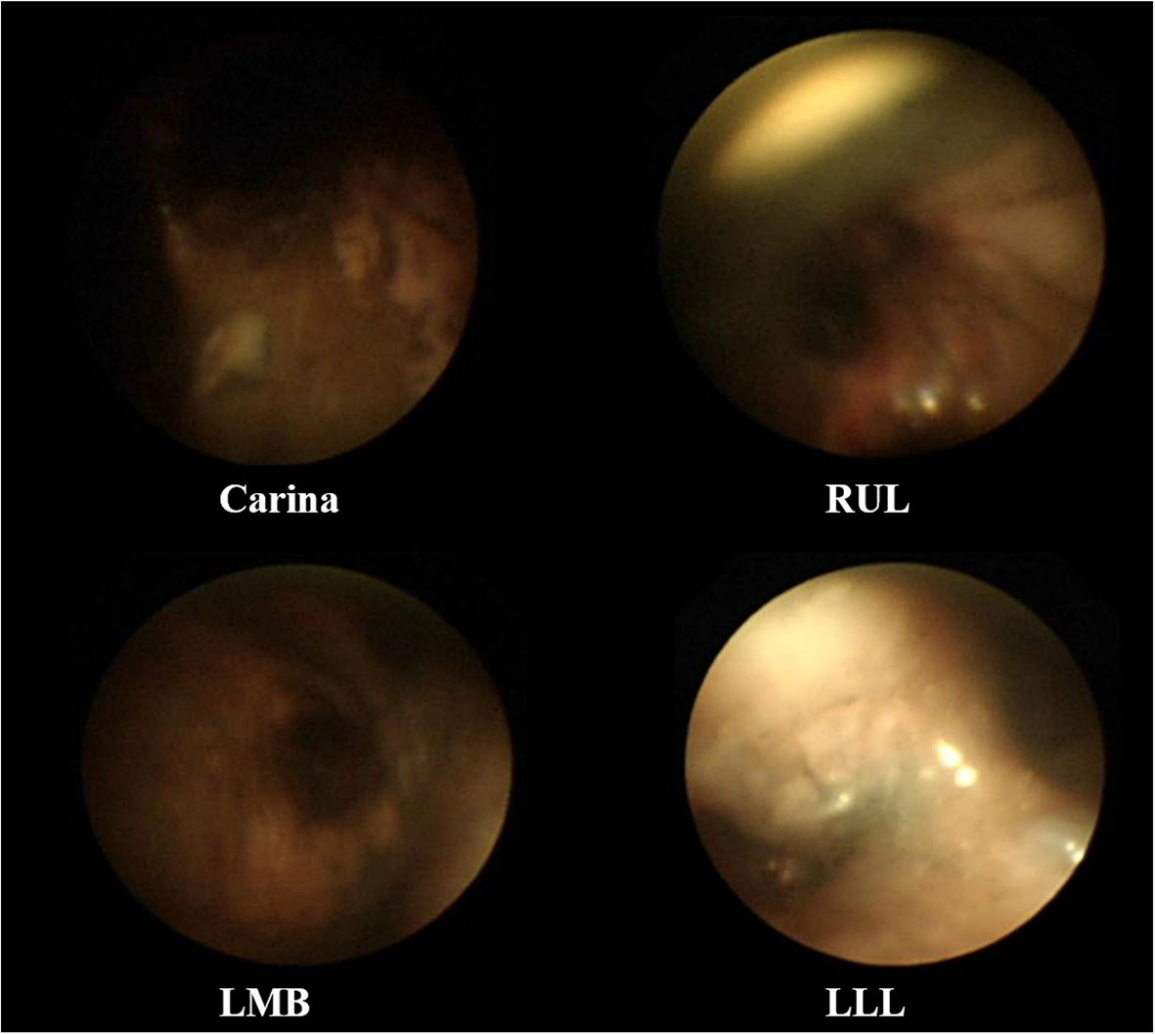
Fiber-optic bronchoscopy images immediately after injury. Airway mucosal swelling or narrowing is not observed but soot can be seen in the carina, right main bronchus, left main bronchus and right upper lobal bronchus. RUL right upper lobe, LMB left main bronchus, LLL left lower lobe

Upon arrival in the ICU, the patient underwent bronchoscopy by a pulmonologist. On FOB, no airway mucosal swelling or narrowing was observed; however, much soot were found in the carina, right main bronchus, left main bronchus, and right upper lobal bronchus (Fig. 3). During bronchoscopy, the pulmonologist tried using normal saline irrigation to remove the soot to no avail. Salbutamol, N-acetylcysteine nebulizer, budesonide nebulizer, and hydrocortisone 50 mg intravenously were administered twice daily to prevent airway complications [4]. Prophylactic antibiotic treatment was initiated with piperacillin and tazobactam. The patient was positioned with head elevated during mechanical ventilation. There were no respiratory infection source in the culture tests performed during hospitalization. Medical treatments for inhalation burn was continued until day 26, at the time of complete weaning of mechanical ventilation.

**Fig. 4:**
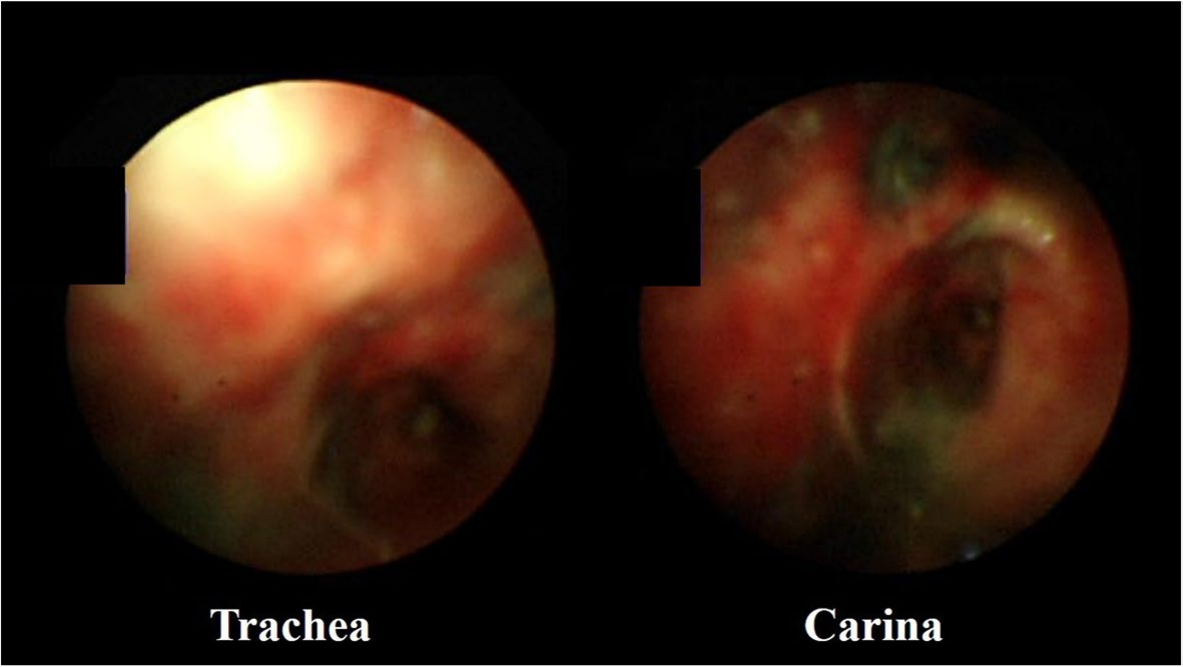
Fiber-optic bronchoscopy image taken on the third day after injury. Airway mucosal erythematous edema can be observed in the trachea and carina

**Fig. 5:**
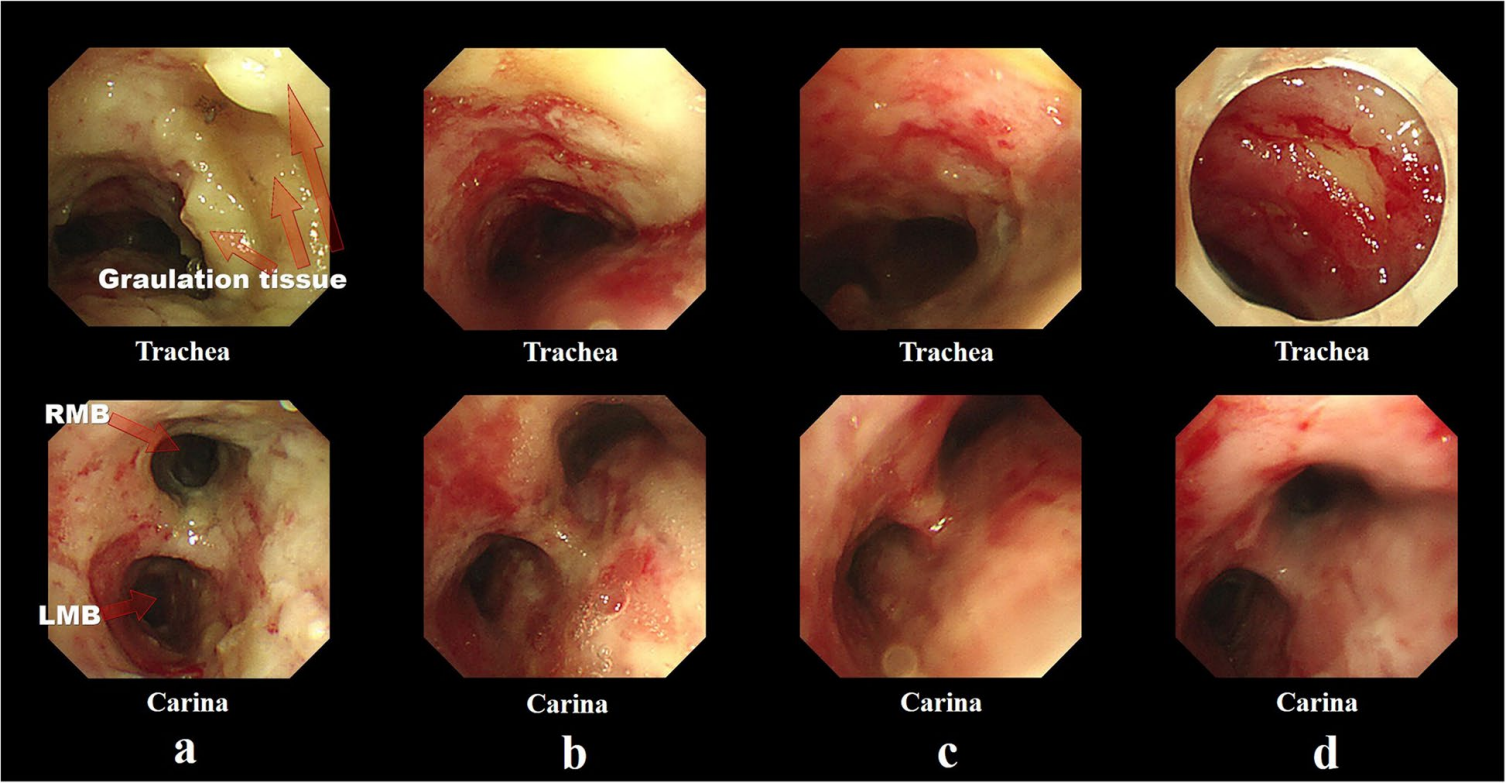
Post-injury fiber-optic bronchoscopy images. **a** On the image taken fourteenth days post-injury, significant granulation tissue and secretion are observed in the distal trachea and carina. **b** On the seventeenth day images, granulation tissue and secretion are reduced compared to before. **c** On the twenty-first day images, the upper respiratory tract epithelium and mucosa can be seen normalizing. **d** On the images taken on the thirty-fourth day, the granulation tissue can no longer be observed, and upper respiratory tract epithelium and mucosa recovery is almost complete. RMB right main bronchus, LMB left main bronchus

On the third day after the inhalation burn event, mucosal erythematous edema was observed on FOB (Fig. 4). Tracheostomy was necessary for the treatment of the patient, and plastic surgery could not be postponed any longer, so we decided to proceed the two surgeries together. On the fourth day, the patient underwent the originally planned plastic surgery and tracheostomy under general anesthesia without any complications. On the sixth day, the first ventilator-weaning trial failed and she was placed on a portable ventilator. Deep breathing and expectorant training were initiated at the bedside. Afterward, daytime ventilator weaning was gradually performed. On the fourteenth day, significant granulation tissue and secretion were observed in the distal trachea and carina (Fig. 5a). On the seventeenth day, the mucus was dried in the trachea, and granulation tissue was reduced compared to prior observations (Fig. 5b). By the twenty-first day, the upper respiratory tract epithelium and mucosa had normalized (Fig. 5c). The ventilator was completely weaned on the twenty-fourth day. On the twenty-sixth day, she was transferred to the Department of Rehabilitation Medicine for swallowing rehabilitation and rehabilitation for delirium that occurred during hospitalization. On the thirty-fourth day, granulation tissue was no longer observed, and upper respiratory tract epithelium and mucosa recovery were almost complete (Fig. 5d). On the thirty-eighth day, the patient underwent tracheostomy closure surgery under local anesthesia. She was discharged from the hospital 40 days after the burn event.

## Discussion and conclusions

This was a case of a breathing circuit fire caused by an unexpected problem with an electrical appliance. A BCW provides heated and humidified anesthetic gases by heating the distilled water inside the circuit [3]. A heating system exists inside the breathing circuit and is wired to the outside controller and supplied with electric power. There are two types of wires in this system: a temperature sensor wire and a heating wire, and they are operated automatically while maintaining the set temperature. After a thorough manufacturer’s investigation of the damaged circuit, they found that both the sensor and heating wires were damaged, which implies an electrical short circuit between the two wires led to the fire. Although a small current always flows through the temperature sensor wire, an electric short circuit causes an overcurrent flow. This phenomenon became the ignition source in our case, which was reproduced and confirmed by the manufacturer’s post-accident investigation. The medical staff first explained to the patient’s family about the possibility of a fire caused by a BCW defect. After post-accident investigation, the manufacturer directly explained this to the patient and patient’s family again. To prevent the same accident, the manufacturer decided to provide all BCW after trial use. In addition to the ignition source, the high concentration of oxygen in the BCW and the plastic breathing circuit served as fuel for the fire. At FiO_2_ > 25%, airway fires are possible, and we supplied a mixture of gases with an FiO_2_ of 50% [5]. Even if there were no BCW-related accidents before this fire, we always have to be aware of the fire hazards associated with electrical appliances.

**Fig. 6:**
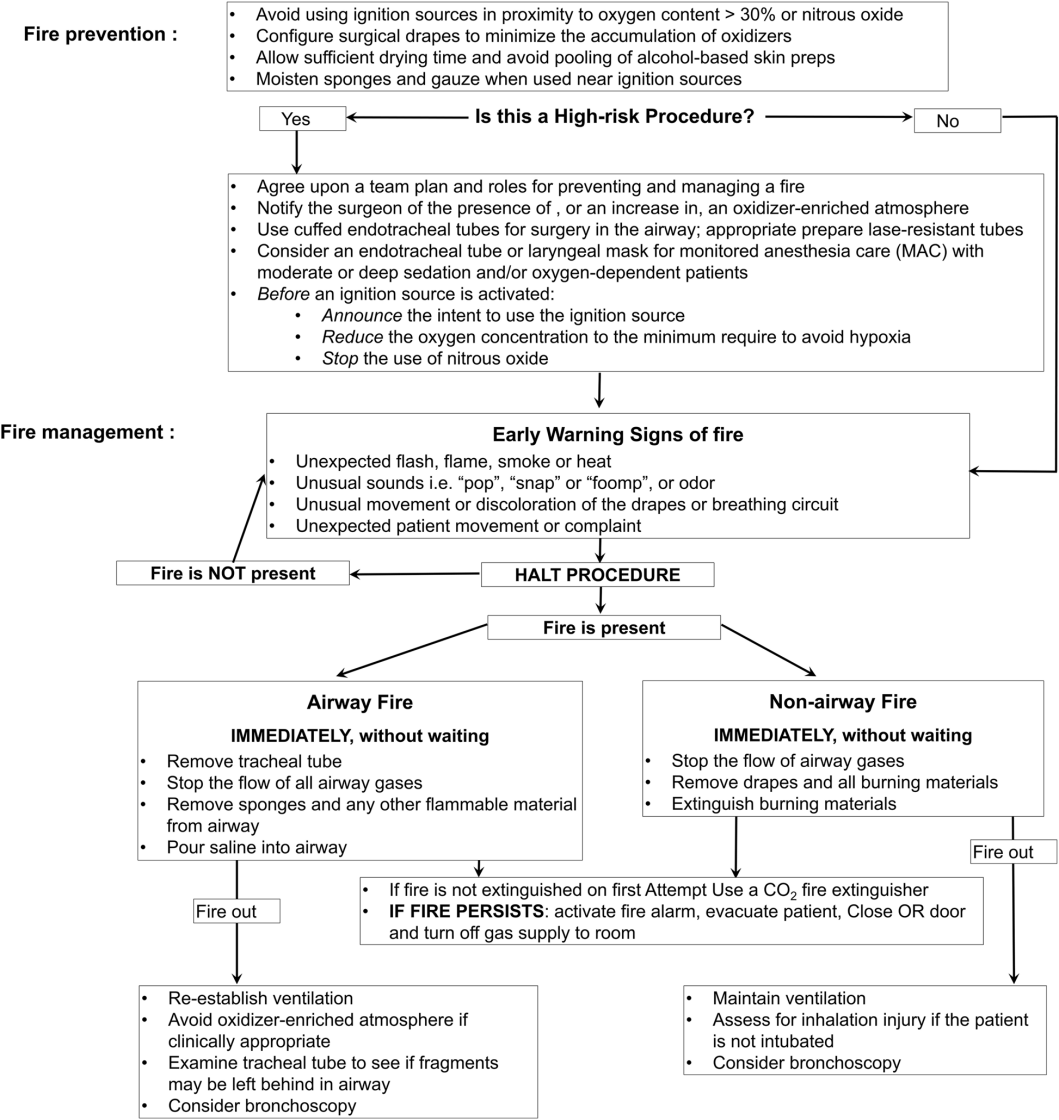
Operating room fire prevention and management algorithm. Revised algorithm from the 2013 American Society of Anesthesiologists Practice Advisory on the Prevention and Management of Operating Room Fires

Anesthesia-related OR fires come in direct contact with the patient’s respiratory system and can be fatal [6, 7]. In our patient, a serious inhalation burn occurred with only 2–3 breaths within 10 s of the fire breaking out. Fortunately, our patient recovered without any long-term complications, although the recovery took 40 days. In the event of an OR fire, there were algorithms for prevention and management (Fig. 6) [8]. When a fire occurs around the airway, ventilation must be stopped immediately, and the ETT should be disconnected from the anesthesia machine to cut off the oxygen supply and prevent further inhalation burns [9, 10]. The removal of the oxygen source and ETT should occur simultaneously. Additionally, physicians should consider washing the airways with cold water but should be aware of the possibility of aspiration of foreign objects. The patient was checked for lung damage due to heat or smoke inhalation through bronchoscopy, chest imaging, SpO_2_ monitoring for more than 24 h, and arterial blood gas analysis; long-term tracheal intubation was considered when damage was confirmed. Airway obstruction due to mucosal edema usually peaks 24 h post-burn [1, 11].

There are some precautions that we must implement for the prevention of fire or management of patients. First, we must always verify that all medical electric appliances work properly before using them directly on a patient. If we had checked the BCW function before application to the patient, it would not have caused such damage to the patient, even though the fire could not have been prevented. Second, after separating the ETT from the breathing circuit, we must immediately replace it with a new ETT. As we performed bag-valve mask ventilation without replacement of the ETT, soot inside the trachea likely entered further into the airway. Soot acts as an inflammatory substance in the airways and causes burns inside the respiratory system [11, 12]. Third, the trachea and bronchi must be immediately washed after the fire with saline to reduce the burns caused by the remaining high-temperature air [8]. Herein, an FOB with a suction function may be useful because there is a possibility of foreign material aspiration. The FOB initially used in the OR had no suction function; therefore, we were unable to perform cold normal saline irrigation.

In conclusion, this is the first report of a fire caused by BCW. The aim of our case report is to share our experience of how we responded to an airway-related fire in an OR and treated the patient. It cannot be overemphasized that the electrical medical appliance associated with the airways are fatal to the patient in the event of a fire, so caution should always be exercised.

## Data Availability

All data related to this case report are contained within the manuscript.

## Abbreviations

BCW: Breathing circuit warmer
OR: Operating room
ETT: Endotracheal tube
HR: Heart rate
BP: Blood pressure
SpO2: Blood oxygen saturation
FiO2: Fraction of inspired oxygen
FOB: Fiber-optic bronchoscopy
ICU: Intensive care unit
